# Serum Neurofilament Light Chain in Multiple Sclerosis: Superiority of Age‐ and BMI‐Corrected *Z* Scores/Percentiles Over Absolute Cutoff Values for Prediction of Treatment Response

**DOI:** 10.1002/acn3.70149

**Published:** 2025-08-03

**Authors:** Maximilian Einsiedler, Aleksandra Maleska Maceski, Sofia Sandgren, Johanna Oechtering, Sabine Schaedelin, Lisa Hofer, Amar Zadic, Juan Francisco Vilchez Gomez, Lester Melie‐Garcia, Alessandro Cagol, Riccardo Galbusera, Sebastian Finkener, Patrice Lalive, Marjolaine Uginet, Stefanie Müller, Caroline Pot, Amandine Mathias, Renaud Du Pasquier, Robert Hoepner, Andrew Chan, Giulio Disanto, Chiara Zecca, Marcus D’Souza, Lars G. Hemkens, Tobias Derfuss, Özgür Yaldizli, Patrick Roth, Claudio Gobbi, David Brassat, Björn Tackenberg, Henrik Zetterberg, Tjalf Ziemssen, Heinz Wiendl, Klaus Berger, Marco Hermesdorf, Fredrik Piehl, Ludwig Kappos, Cristina Granziera, Ahmed Abdelhak, David Leppert, Eline A. J. Willemse, Pascal Benkert, Jens Kuhle, Swiss Multiple Sclerosis Cohort Study (SMSC)

**Affiliations:** ^1^ Multiple Sclerosis Centre and Research Center for Clinical Neuroimmunology and Neuroscience (RC2NB), Neurology, Departments of Biomedicine and Clinical Research University Hospital and University of Basel Basel Switzerland; ^2^ Department of Clinical Neuroscience, Institute of Neuroscience and Physiology at Sahlgrenska Academy University of Gothenburg Gothenburg Sweden; ^3^ Sahlgrenska University Hospital, Department of Neurology Region Västra Götaland Gothenburg Sweden; ^4^ Translational Imaging in Neurology (ThINK) Basel, Department of Biomedical Engineering, Faculty of Medicine University Hospital Basel and University of Basel Basel Switzerland; ^5^ Department of Neurology Cantonal Hospital Aarau Aarau Switzerland; ^6^ Department of Clinical Neurosciences, Division of Neurology, and Department of Medicine Translational Biomarker Group Geneva Switzerland; ^7^ Diagnostic Department, Division of Laboratory Medicine Geneva University Hospital Geneva Switzerland; ^8^ Department of Pathology and Immunology, Faculty of Medicine University of Geneva Geneva Switzerland; ^9^ Department of Neurology Cantonal Hospital St. Gallen St. Gallen Switzerland; ^10^ Service of Neurology and Laboratories of Neuroimmunology, Department of Clinical Neurosciences Lausanne University Hospital (CHUV) and University of Lausanne Lausanne Switzerland; ^11^ Department of Neurology, Inselspital, Bern University Hospital University of Bern Bern Switzerland; ^12^ Multiple Sclerosis Center, Neurocenter of Southern Switzerland EOC Lugano Switzerland; ^13^ Faculty of Biomedical Sciences Università della Svizzera Italiana (USI) Lugano Switzerland; ^14^ Department of Neurology and Clinical Neuroscience Center University Hospital Zurich and University of Zurich Zurich Switzerland; ^15^ Novartis Pharma AG Basel Switzerland; ^16^ Department of Neurology, Hôpital de la Pitié‐Salpêtrière AP‐HP Paris France; ^17^ Department of Neurology Philipps‐University Marburg Germany; ^18^ F. Hoffmann‐La Roche Basel Switzerland; ^19^ Department of Psychiatry and Neurochemistry, Institute of Neuroscience and Physiology the Sahlgrenska Academy at the University of Gothenburg Mölndal Sweden; ^20^ Clinical Neurochemistry Laboratory Sahlgrenska University Hospital Mölndal Sweden; ^21^ Department of Neurodegenerative Disease UCL Institute of Neurology, Queen Square London UK; ^22^ UK Dementia Research Institute at UCL London UK; ^23^ Hong Kong Center for Neurodegenerative Diseases InnoHK Hong Kong China; ^24^ Wisconsin Alzheimer's Disease Research Center, University of Wisconsin School of Medicine and Public Health University of Wisconsin‐Madison Madison WI USA; ^25^ Center of Clinical Neuroscience, Department of Neurology, University Hospital Carl Gustav Carus, Dresden Technical University of Dresden Dresden Germany; ^26^ Department of Neurology and Neurophysiology University Hospital Freiburg Freiburg Germany; ^27^ Institute of Epidemiology and Social Medicine University of Münster Münster Germany; ^28^ Department of Clinical Neuroscience Karolinska Institutet Stockholm Sweden; ^29^ Center for Neurology, Academic Specialist Center Stockholm Health Services Stockholm Sweden; ^30^ Department of Neurology and Weill Institute for Neurosciences University of California San Francisco CA USA

**Keywords:** multiple sclerosis, neurofilament, prediction, *Z* scores

## Abstract

**Objective:**

Prognostication of disease course and prediction of treatment response in multiple sclerosis is an unmet need. We compared the performance of serum neurofilament light chain *Z* scores (age‐ and BMI‐adjusted) with absolute concentrations for the prediction of response to disease‐modifying therapy.

**Methods:**

Observational cohort study including the first serum sample of participants after the start of fingolimod therapy. We estimated hazard ratios for future relapses comparing participants with high (upper quartile) versus lower neurofilament light chain levels, based on either absolute concentration or *Z* score cutoffs. We compared the prognostic/predictive performance of these two measures for the occurrence of new/enlarging T2w lesions in longitudinal MRI.

**Results:**

We included 447 participants (median [IQR] age, 41.3 [32.1–49.2] years; 65.1% female); median follow‐up 8.3 years [6.0–10.3]. Participants with a high neurofilament light chain *Z* score (*Z* ≥ 1.2/88.5 percentile) were more likely to experience future relapses (HR: 1.80, 95% CI 1.27–2.54, *p* < 0.001) compared to those below this threshold while this dichotomy could not be demonstrated with absolute concentration cutoffs (≥ vs. < 10.8 pg/mL; HR: 0.94, 95% CI 0.64–1.38, *p* = 0.75). Furthermore, patients with upper quartile *Z* scores were associated with a higher incidence of new/enlarging T2w lesions compared with those below this threshold (OR: 1.88, 95% CI 1.31–2.70, *p* < 0.001); again, absolute concentration cutoffs failed to identify this risk (OR: 1.20, 95% CI 0.82–1.77, *p* = 0.34). These findings were confirmed when patients having started alternative oral treatments were also included (*n* = 713).

**Interpretation:**

Serum neurofilament light chain *Z* scores consistently outperformed absolute concentration cutoffs for prognostication of clinical/radiological disease activity and may facilitate individual prediction of treatment response.

## 1 Introduction

Neurofilament light chain is a cytoskeletal protein of neurons and an increasingly used marker of neuro‐axonal damage across many acute and chronic neurological diseases [1]. In multiple sclerosis (MS), serum neurofilament light chain (sNfL) concentration correlates with disease activity, response to disease modifying therapy (DMT) and disability accumulation [2, 3].

Several immunoassay platforms have been developed for the quantification of sNfL, where levels are typically reported as absolute concentration in pg/mL, without systematic correction for confounding factors recognized in recent years. Physiologically, sNfL levels increase with age [4, 5] and decrease with higher body mass index (BMI) [6], the first being particularly pronounced above 50 years of age [7]. Even though, on a group level, high absolute sNfL concentrations are clearly associated with worse long‐term disability outcomes [8], the lack of a tool to correct for these physiological factors has limited the interpretability of individual measurements. Inherently, the use of fixed absolute cutoffs to define pathological levels of sNfL neglects these factors and is a conceptual source of inaccuracy for the use of sNfL in personalized medicine.

To overcome this gap, we have recently established age‐ and BMI‐corrected percentiles and *Z* scores for sNfL from large reference databases of participants with no evidence of central nervous system (CNS) disease, to provide normative data in adults and children [7, 9].

Already subtle increases of sNfL *Z* scores or percentiles can capture subclinical disease activity, anticipate the future course of disease, and allow monitoring treatment response beyond clinical and magnetic resonance imaging (MRI) measures in patients with MS [7].

However, a direct comparison of concentrations of absolute value versus (vs) *Z* score cutoffs for the definition of elevated sNfL levels has not been executed.

In this study, we compare the performance of these two measures of sNfL for the prediction of relapse and MRI activity in individual MS patients after having started fingolimod therapy.

## 2 Methods

### 2.1 Study Design, Study Population, and Clinical Endpoint

The study was conducted in participants of the Swiss Multiple Sclerosis Cohort (SMSC), a prospective observational study collecting clinical and radiological data as well as serum samples across eight MS centers in Switzerland [10].

We included all participants with relapsing MS who had started a first treatment with fingolimod (or in a sensitivity analysis adding patients who had started with dimethyl fumarate, diroximel fumarate, ozanimod, siponimod, or teriflunomide) since 2012. The first sample under treatment (median 1 year after DMT start) was defined as the index sample and used to assess treatment response. Relapses were defined as new, worsening, or recurrent neurologic symptoms, in the absence of fever, infections, or adverse reactions to prescribed medications, that lasted ≥ 24 h and were preceded by a stable or improving neurologic status of ≥ 30 days [11]. This study followed the ‘Strengthening the Reporting of Observational Studies in Epidemiology’ (STROBE) reporting guidelines [12].

### 2.2 sNfL Measurements

All serum samples were acquired according to standard procedures and stored at −80°C [13]. sNfL concentrations were measured using the Neurology 2‐plex B Single molecule array (Simoa) assay (Quanterix, Billerica, MA) according to the manufacturer's instructions on the Simoa HD‐X platform. Age‐ and BMI‐adjusted sNfL *Z* scores were obtained as previously described [7] and express the deviation of sNfL from values in the reference population of healthy controls in terms of number of standard deviations from the mean. Percentiles express the percentage of healthy controls expected to have a sNfL value (adjusted for age and BMI) lower than the considered value. *Z* scores and percentiles are interchangeable measures (*Z* score 0 = 50th percentile; *Z* score of 1, 2, and 3 correspond to the 84.1st, 97.7th, and 99.9th percentile, respectively) [7].

### 2.3 MRI Protocol and Endpoints

Brain MRIs were performed annually using a standardized protocol, including 3D fluid‐attenuated inversion recovery (FLAIR) and pre‐ and postcontrast T1‐weighted (T1w) sequences with 1 mm^3^ spatial resolution. The evaluated outcomes were T2w hyperintense lesion volume, number of new/enlarging T2w hyperintense lesions (NEL), and number of contrast‐enhancing T1w lesions (CEL). T2w lesion volumes were automatically assessed using a deep learning‐based approach [14], followed by manual quality assessment and correction. We performed a longitudinal analysis of all FLAIR and T1w images using LeMan‐PV [15, 16] to automatically detect NEL. The outputs were then manually reviewed by experienced raters in the central SMSC reading center.

### 2.4 Statistical Analysis

#### 2.4.1 sNfL and MRI Activity

To evaluate associations with radiological activity (CEL, past NEL, and future NEL separately), we included all 6‐ or 12‐monthly longitudinal sNfL samples obtained during the follow‐up and having available corresponding MRI information. We defined past NEL as the occurrence of NEL in the year before sampling, and future NEL as the occurrence of NEL in the year after sampling. The association between biomarker level and MRI activity was assessed in two ways: (1) Using sNfL as the dependent variable, we investigated how biomarker levels (continuous absolute sNfL or *Z* scores) differed at time points with versus without concurrent, past, or future MRI activity. (2) Using MRI activity as the dependent variable, we compared whether elevated absolute sNfL levels or *Z* scores (“high” vs. “low”) were associated with an increased risk of MRI activity. For these analyses, to compare cutoffs of absolute values and *Z* scores, we applied a split based on quartiles, with values in quartiles (Q) 1–3 considered “low” and values ≥ Q4 considered “high”.

##### 2.4.1.1 Modeling sNfL Levels (sNfL Levels as Dependent Variable)

sNfL levels (absolute values or *Z* scores) were used as the dependent variable to assess differences across MRI activity groups—specifically, patients with or without CEL, and with or without past or future NEL. These comparisons were visualized using boxplots and analyzed using linear generalized estimating equation (GEE) models to account for repeated measures within patients. For all GEE models, an exchangeable correlation structure was used. Absolute values of sNfL needed to be log‐transformed due to their non‐normal distribution, where the back‐transformed estimates represent multiplicative effects (group difference in percent). This transformation was not needed for Z scores, and the estimated group differences were therefore presented as additive effects (difference in *Z* scores). We performed additional analyses comparing absolute sNfL values versus *Z* scores according to categories of numbers (0; 1–5; 6–10; ≥ 10) of NEL occurring in the following year. To quantify the gain in precision obtained by using *Z* scores, we numerically described the effect sizes using Cohen's *d*.

##### 2.4.1.2 Modeling Risk of MRI Activity (MRI Outcomes as Dependent Variable)

We evaluated the prognostic value of elevated sNfL by modeling MRI outcomes (CEL and future NEL) as dependent variables in univariable logistic GEE models. sNfL (absolute or Z score), dichotomized as “high” versus “low”, served as the independent variable. We reported estimated odds ratios (ORs) with 95% confidence intervals (CIs).

#### 2.4.2 sNfL and Future Relapse Activity

We included all patients with an index serum sample under fingolimod (or in a sensitivity analysis under fingolimod or another oral DMT) available 8 to 24 months after the start of this treatment. Kaplan–Meier analysis was used to study the association between biomarker levels at the index sample and time to future relapse. For absolute concentrations and *Z* scores, we again applied the split based on quartiles, with values in Q 1–3 considered “low” and values ≥ Q4 considered “high”. Cox regression models were used to calculate hazard ratios (HR) for relapses, including 95% confidence intervals. Additionally, Cox regression models were calculated for various cutoff points to validate the robustness of findings irrespective of the chosen cutoff.

The time to event analysis was performed irrespective of treatment switches. Additionally, we performed a sensitivity analysis limited to events under fingolimod by censoring patients 3 months after DMT stop or switch to include potential relapses coinciding with the treatment change. In an additional sensitivity analysis, we evaluated only patients with an age at index sample of ≤ 55 years to evaluate the performances of sNfL *Z* scores in this subgroup where the effect of age on physiological sNfL values is less pronounced.

### 2.5 Ethics

This study was approved by the local ethics committee and all patients gave written informed consent.

## 3 Results

### 3.1 Demographics and Baseline Measures

**FIGURE 1:**
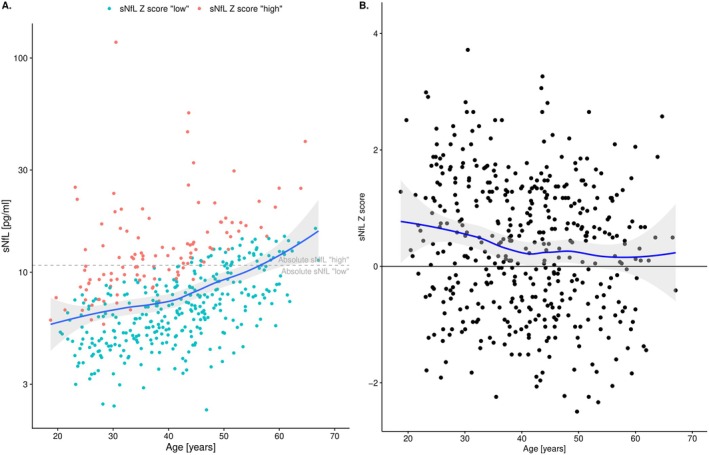
Absolute sNfL concentrations 1 year after fingolimod start and corresponding age‐ and BMI‐adjusted *Z* scores. (A) Absolute sNfL concentrations 1 year after fingolimod start color coded by *Z* score (red: upper quartile of all values (“high”; *Z* score ≥ 1.2); blue: quartiles 1–3 (“low”; *Z* score < 1.2)). The absolute value cutoff (upper quartile of all values: 10.8 pg/mL) is shown by the horizontal dashed line. In younger patients, an absolute cutoff (horizontal line at 10.8 pg/mL) may miss “high” *Z* scores (false negatives: red dots below the line). Conversely, in older patients, “low” *Z* scores (< 1.2) can overlap with absolute values above the cutoff (false positives: blue dots above the line). The nonparametric smoothing line in blue with 95% confidence band (gray area) indicates the steady increase with age. (B) Scatter plot of sNfL *Z* scores along age in the same population. sNfL *Z* scores were higher in younger patients, reflecting higher sNfL levels than in healthy controls. *Z* score of 0 (50. percentile) indicates the mean (age‐ and BMI‐adjusted) sNfL concentration in the healthy controls reference population. BMI, body mass index; sNfL, serum neurofilament light chain.

We included 447 patients with relapsing MS who had started fingolimod treatment. The index sNfL sample was obtained at a median time of 1 (interquartile range (IQR): [0.9–1.3]) year after DMT initiation, and the median sNfL absolute value was 7.8 (5.5, 10.8) pg/mL, while the *Z* score was 0.4 (−0.5, 1.2). Median follow‐up was 8.3 (6.0–10.3) years (Table 1). More than half of the patients either stopped fingolimod therapy (5.8%) or changed to another treatment (47.4%) during follow‐up time (Table 1). Only a few patients had very high (> 20 pg/mL, *n* = 22) sNfL levels and could be readily identified as having high disease activity based on these elevated absolute concentrations; otherwise, elevated levels were hidden by the strong age‐related increase (as indicated by the smoothing line in Figure 1A). However, by using sNfL *Z* scores, a negative association was observed with higher scores in younger patients (i.e., elevated sNfL levels compared to levels in healthy controls), reflecting increased disease activity in this population (Figure 1B).

**TABLE 1:** Patient characteristics at start of fingolimod therapy.

	All patients (*n* = 447)
Sex (female)	291 (65.1)
Age, years	41.3 [32.1, 49.2]
EDSS	2.0 [1.5, 3.0]
Disease duration, years	7.8 [3.1, 14.1]
Relapse < 3 months before treatment start	97 (21.7)
Treatment start to index sample, years	1.0 [0.9, 1.3]
Follow‐up duration after index sample, years	8.3 [6.0, 10.3]
At index sample	
sNfL (pg/mL)	7.8 [5.5, 10.8]
sNfL *Z* score	0.4 [−0.5, 1.2]
Change in DMT during follow‐up	238 (53.2)
HET	134 (30.0)
Oral	69 (15.4)
Platform	9 (2.0)
Untreated	26 (5.8)

*Note:* Variables are expressed as *n* (%) or median [IQR].

Abbreviations: DMT, disease modifying treatment; EDSS, Expanded Disability Status Scale; HET, high efficacy treatment (alemtuzumab, natalizumab, ocrelizumab, ofatumumab, rituximab); IQR, interquartile range; Oral, oral DMT (cladribine, dimethyl fumarate, diroximel fumarate, ozanimod, siponimod, teriflunomide); Platform, platform DMT (interferon beta 1a and b and glatiramer acetate); sNfL, serum neurofilament light chain.

### 3.2 Cutoffs for Absolute Values and *Z* Scores of sNfL

“High” sNfL levels were defined as ≥ 10.8 pg/mL (*n* = 112) in absolute values, or a *Z* score of ≥ 1.2 (*n* = 114), to identify a similarly sized proportion of the study cohort. 44/112 (39%) patients with “high” sNfL levels based on absolute value had a “low” (i.e., < 1.2) *Z* score, meaning that these would be classified as false positives for “pathologic” considering confounder‐adjusted *Z* scores as the gold standard (Figure 1). In return, 14% (46/335) of patients with “low” absolute sNfL had increased *Z* scores and would escape identification for having pathologically increased sNfL levels according to *Z* score, that is, being false negatives based on absolute values. Overall, 20% (90/447) of patients showed discordant results between these two definitions of “high” sNfL values (Table S1).

### 3.3 Relation of Absolute Values and *Z* Scores of sNfL With MRI Activity

#### 3.3.1 Contrast‐Enhancing T1w Lesions (CEL)

MRI timepoints with CEL (3%; 49/1535 available MRI timepoints), as compared to those without CEL, showed 53% higher absolute sNfL levels (estimate (est.): 1.53; 95% CI 1.27–1.85; *p* < 0.001) and, on average, 0.95 units higher sNfL *Z* scores (95% CI 0.63–1.26; *p* < 0.001; Figure S1). Application of sNfL *Z* scores versus absolute sNfL concentrations increased the magnitude of difference between the groups (as expressed by the effect size measure Cohen's *d*: 1.33 vs. 0.88). Figure S1 reveals yet another feature: a considerable number of patients without CEL had sNfL values above the Q4 of patients having CEL, either with absolute values or *Z* scores, indicative of ongoing disease activity.

**FIGURE 2:**
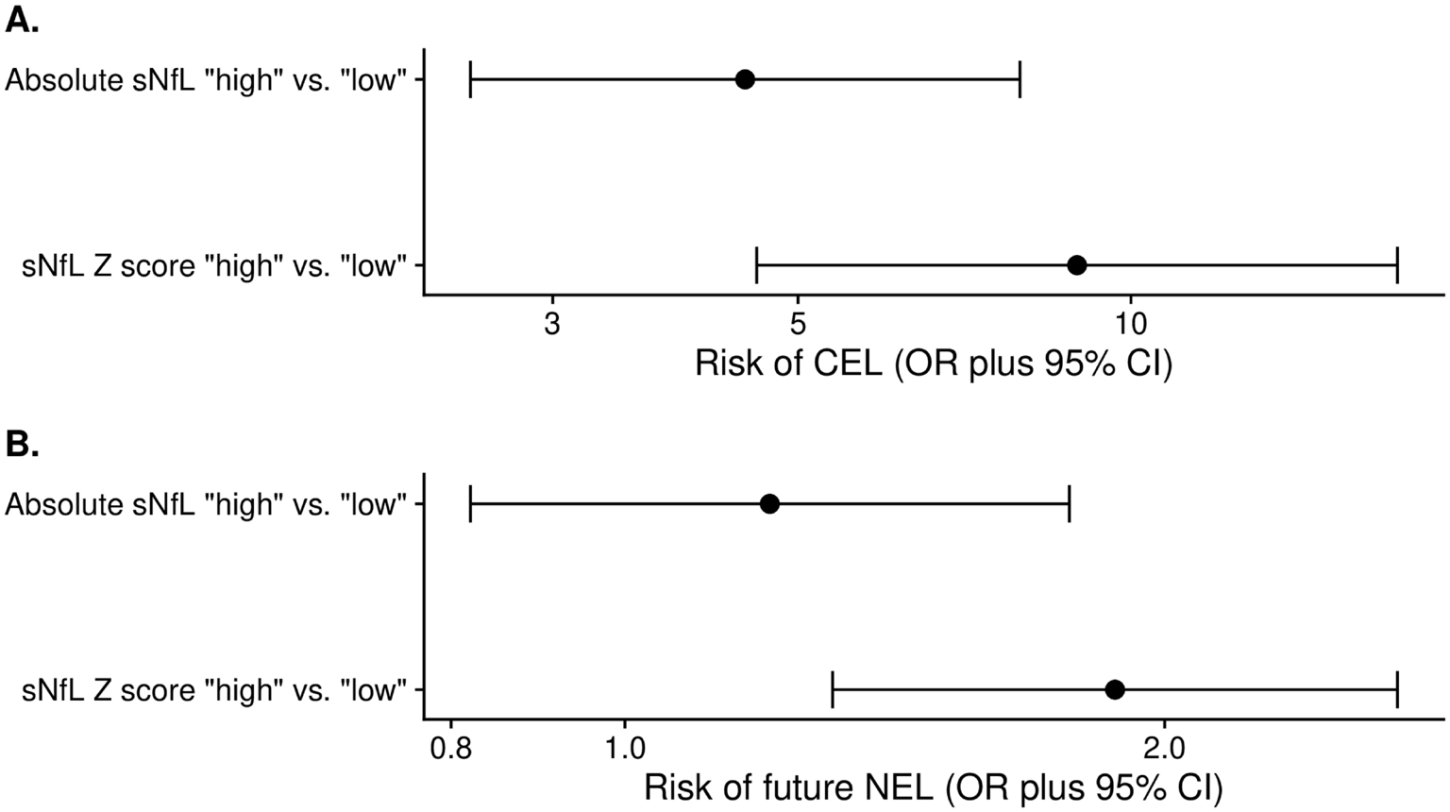
Risk of MRI activity predicted by elevated absolute sNfL values versus age‐ and BMI‐adjusted *Z* scores. (A) Patients with a “high” sNfL *Z* score (Q4) had a higher risk of concurrent CEL compared to patients with “low” sNfL *Z* scores (OR 8.94, 95% CI 4.59–17.42, *p* < 0.001), the effect being less pronounced with absolute values (OR 4.48, 95% CI 2.53–7.94, *p* < 0.001). (B) Patients with a “high” versus “low” sNfL *Z* score had a higher risk of future NEL (OR 1.88, 95% CI 1.31–2.70, *p* < 0.001). No difference was seen using absolute sNfL values (OR 1.20, 95% CI 0.82–1.77, *p* = 0.34). Estimates from univariable logistic GEE models with sNfL as independent variable and radiological activity (respectively CEL and future NEL) as binary outcome. BMI, body mass index; CEL, contrast‐enhancing T1w lesion; CI, confidence interval; GEE, generalized estimating equation; MRI, magnetic resonance imaging; NEL, new/enlarging T2w lesions; sNfL, serum neurofilament light chain; OR, odds ratio; T1/T2w, T1/T2‐weighted; Q, quartile.

A logistic regression model with the presence of CEL as the dependent variable confirmed the strong association with elevated sNfL and allowed for a numerical comparison of absolute and *Z* score cutoffs based on quartiles: Patients with “high” absolute values of sNfL were at higher risk of concomitant CEL compared with patients with “low” sNfL values (OR 4.48, 95% CI 2.53–7.94, *p* < 0.001); however, based on *Z* scores, this association was more pronounced (OR 8.94, 95% CI 4.59–17.42, *p* < 0.001, Figure 2A).

#### 3.3.2 New/Enlarging T2w Lesions (NEL)

In patients with past NEL (21.5%; 193/899 available time points), absolute sNfL values were 15% higher compared with patients with stable MRI (8.5 pg/mL [6.1, 12.5] vs. 7.6 pg/mL [5.6, 10.4], est. 1.15, 95% CI 1.07–1.23, *p* < 0.001). Again, this difference was more pronounced with sNfL *Z* scores (0.67 [−0.25, 1.48] vs. 0.08 [−0.73, 0.71], est. +0.45 *Z* score units, 95% CI 0.28–0.61, *p* < 0.001) (Figure S2A), which is also reflected by respective effect sizes (Cohen's *d* = 0.28 vs. *d* = 0.52).

Absolute sNfL values did not differ between patients with future NEL (21.8%; 191/878 available timepoints) versus those with stable MRI (8.1 pg/mL [6.0, 11.3] vs. 7.5 [5.6, 10.5], est. 1.06, 95% CI 0.99–1.13, *p* = 0.11), while *Z* scores clearly differentiated between the groups (0.64 [−0.18, 1.36] vs. 0.13 [−0.71, 0.84], est. +0.26 *Z* score units, 95% CI 0.09–0.44, *p* = 0.004); again reflected by higher effect sizes favoring the use of *Z* scores (Cohen's *d* = 0.44 vs. *d* = 0.22) (Figure S2B).

**FIGURE 3:**
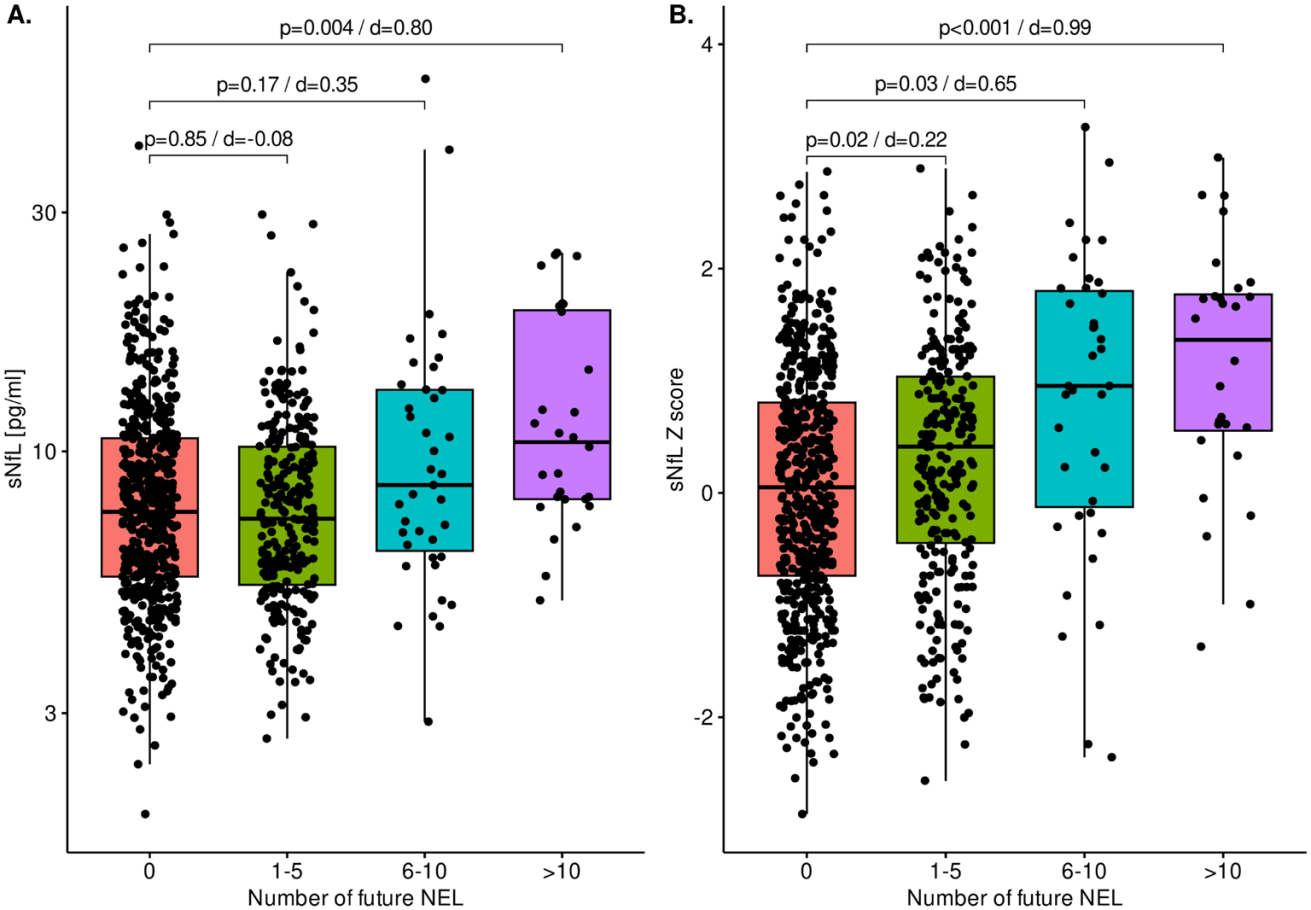
Differences in absolute values and in age‐ and BMI‐adjusted *Z* scores of sNfL according to the number of future new/enlarging T2w lesions (NEL). (A) Absolute values: Levels were similar between patients without and 1–5 NEL (est. 1.00, 95% CI 0.96–1.05, *p* = 0.85, *d* = −0.08). Levels were increased by 14% in patients with 6–10 NEL (est. 1.14, 95% CI 0.95–1.37, *p* = 0.17, *d* = 0.35), and by 32% in patients with > 10 NEL (est. 1.32, 95% CI 1.09–1.59, *p* = 0.004, *d* = 0.80) compared to patients without NEL. (B) *Z* scores: Levels were increased by 0.15 *Z* score units in patients with 1–5 NEL (est. 0.15, 95% CI 0.02–0.28, *p* = 0.02, *d* = 0.22), by 0.49 *Z* score units with 6–10 NEL (95% CI 0.05–0.93, *p* = 0.03, *d* = 0.65), and by 0.86 *Z* score units with > 10 NEL (95% CI 0.41–1.30, *p* < 0.001, *d* = 0.99), compared to patients without NEL. Application of sNfL *Z* scores versus absolute sNfL concentrations increased the magnitude of difference (as expressed by the effect size measure Cohen's *d*). Group differences are compared statistically using linear GEE models with biomarker level as the dependent variable (absolute sNfL was log‐transformed). BMI, body mass index; CI, confidence interval; GEE, generalized estimating equation; MRI, magnetic resonance imaging; NEL, new/enlarging T2w lesions; sNfL, serum neurofilament light chain; T2w, T2‐weighted.

Only patients with > 10 future NEL had significantly higher absolute sNfL values compared to patients with no NEL (32% higher, est. 1.32, 95% CI 1.09–1.59, *p* = 0.004) (Figure 3). In contrast, using sNfL *Z* scores, elevated levels were detectable even in patients with 1–5 future NEL, with an estimated increase of 0.15 *Z* score units (95% CI 0.02–0.28, *p* = 0.02) resulting in larger effect sizes (Cohen's d) across all groups.

Elevated sNfL prognosticates future NEL: Patients with a “high” sNfL *Z* score had a 1.88‐fold higher risk of having future NEL compared to patients with “low” sNfL *Z* scores (OR 1.88, 95% CI 1.31–2.70, *p* < 0.001, Figure 2B); absolute sNfL values failed to provide this discrimination (“high” vs. “low”: OR 1.20, 95% CI 0.82–1.77, *p* = 0.34).

### 3.4 Predictive Capacity of Absolute Values Versus *Z* Scores of sNfL for Future Relapses

**FIGURE 4:**
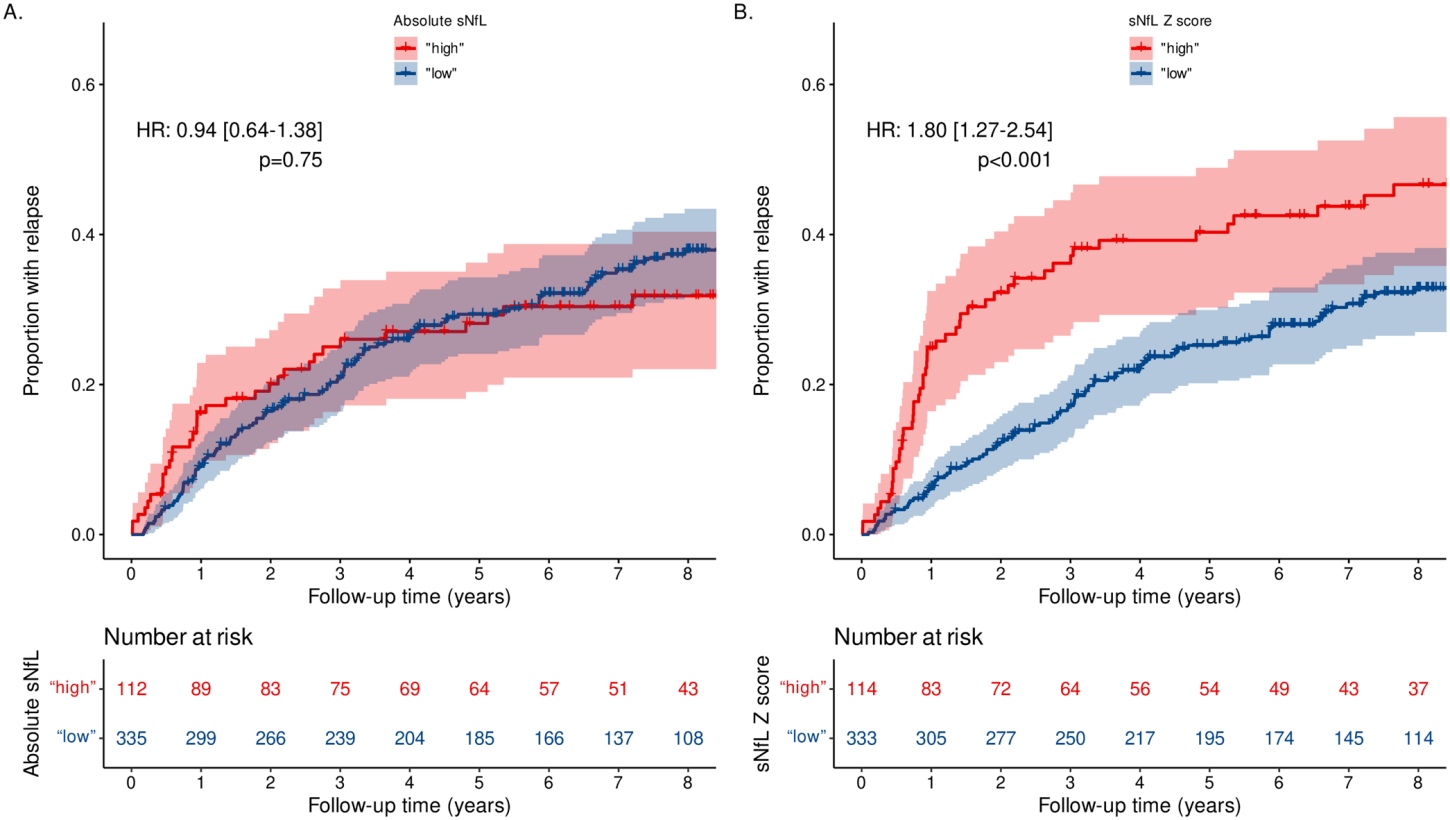
Time to first relapse depending on sNfL, 1 year after fingolimod start using either absolute values or age‐ and BMI‐adjusted *Z* scores. (A) Time to first relapse comparing “high” (Q4; ≥ 10.8 pg/mL) versus “low” (Q1–3; < 10.8 pg/mL) absolute values. (B) Time to first relapse comparing “high” (Q4; ≥ 1.2) versus “low” (Q1–3; < 1.2) *Z* scores. *Z* score cutoffs provided superior predictive accuracy for relapses compared to absolute values (absolute values: HR: 0.94, 95% CI 0.64–1.38, *p* = 0.75; *Z* scores: HR: 1.80, 95% CI 1.27–2.54, *p* < 0.001). The estimated proportion of patients with a relapse in the first 2 years of follow‐up was 32.2% (95% CI 23.0–40.4) in patients with a *Z* score ≥ 1.2 versus 12.3% (95% CI 8.6–15.8) in patients with a *Z* score below that cutoff (Table S2). BMI, body mass index; CI, confidence interval; HR, hazard ratio; Q, quartile; sNfL, serum neurofilament light chain.

Patients with “high” *Z* score were at 1.8‐fold higher risk of future relapse activity (HR: 1.80, 95% CI 1.27–2.54, *p* < 0.001) (Figure 4). This association was not apparent using the absolute sNfL level cutoff (HR: 0.94, 95% CI 0.64–1.38, *p* = 0.75). The estimated proportion of patients with a relapse in the first 2 years of follow‐up after sampling for treatment response was 32.2% (95% CI 23.0–40.4) in patients with a *Z* score ≥ 1.2 versus 12.3% (95% CI 8.6–15.8) in patients with a *Z* score below this cutoff (Table S2).

In a larger cohort (*n* = 713) that encompassed patients under various oral treatments (fingolimod, dimethyl fumarate, teriflunomide, diroximel fumarate, ozanimod, or siponimod) (Table S3) findings in the fingolimod only group were confirmed: patients with “high” sNfL *Z* score were at 1.6‐fold higher risk of future relapses (HR: 1.59, 95% CI 1.18–2.13, *p* = 0.002), Figure S3 compared to those with “low” *Z* scores. Again, this could not be shown using absolute sNfL concentrations (HR: 0.78, 95% CI 0.55–1.1, *p* = 0.16).

**FIGURE 5:**
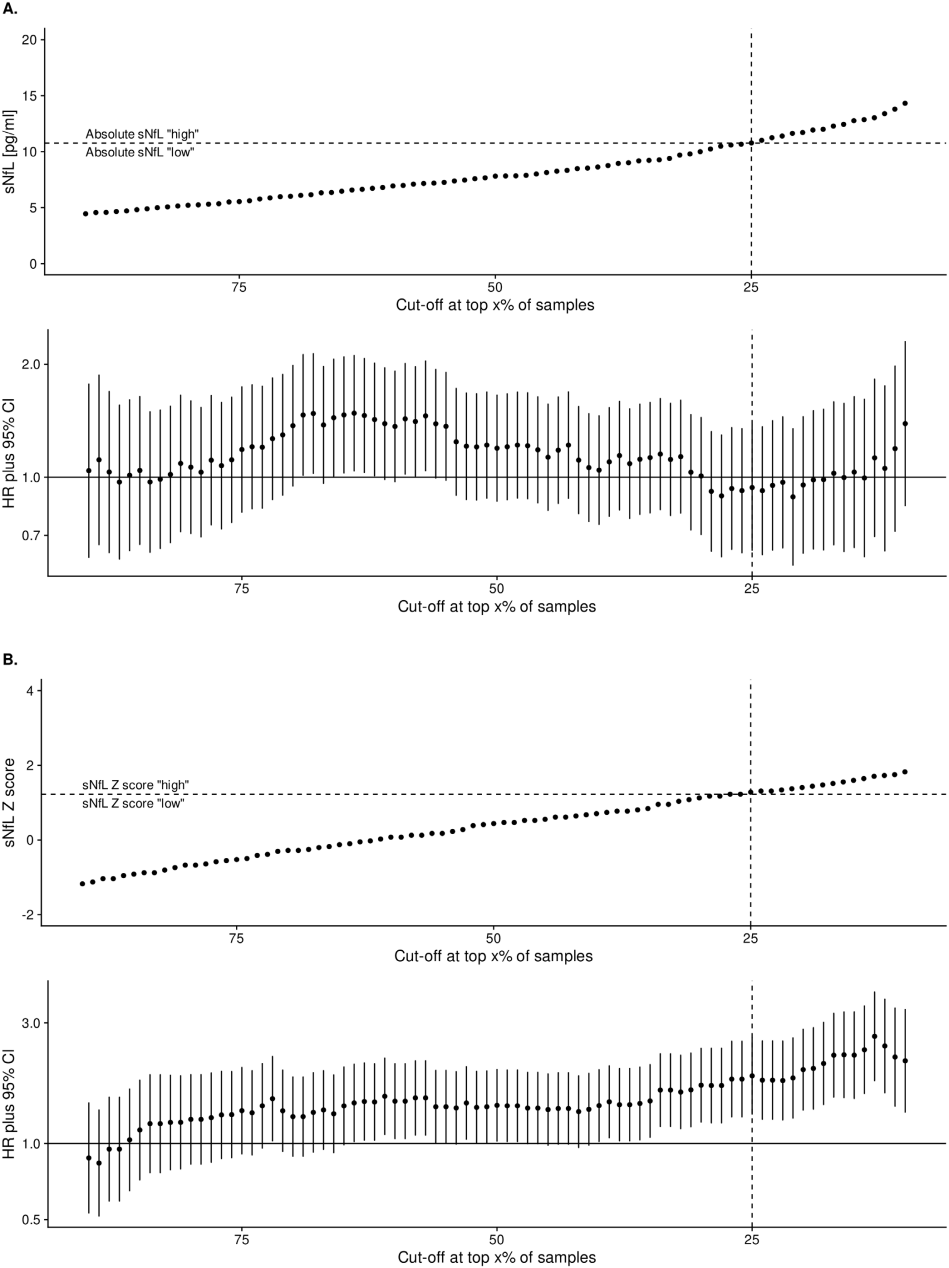
Impact of different cutoffs for (A) absolute values and (B) *Z* scores of sNfL for the prediction of future relapses. Top panels: Dependence of the proportion of samples considered “high” when increasing sNfL absolute level (A) and *Z* score (B) cutoff levels. In this study, the standard cutoff was set at the top 25% of samples, that is, the fourth quartile (dashed vertical line). This corresponds to a cutoff of 10.8 pg/mL in absolute values (A) and a *Z* score of 1.2 (B), represented by the respective dashed horizontal lines. Bottom panels: Resulting hazard ratios for future relapses (dots) with 95% confidence intervals (vertical lines) estimated by individual Cox model for each potential cutoff dichotomizing in “high” and “low” sNfL. Absolute sNfL cutoffs (A) were never predictive of future relapses, while most of the higher sNfL *Z* score cutoffs (i.e., above Z: 0.8, B) were significantly predictive of future relapses (i.e., 95% CI interval not crossing 1.0). CI, confidence interval; HR, hazard ratio; sNfL, serum neurofilament light chain.

We then compared the sensitivity of absolute values versus *Z* scores for the prognosis and prediction of future relapses when using a continuum of cutoffs (Figure 5). The impact on the relapse HR of the two types of cutoffs showed that sNfL *Z* score cutoffs > 0.8 were consistently prognostic of relapses, while absolute values were not for any chosen threshold.

In a sensitivity analysis censoring patients who stopped or switched to another DMT during follow‐up, a similar difference in prognostic performance was found (patients with a “high” *Z* score were at more than 2‐fold higher risk of future relapses (HR: 2.10, 95% CI 1.43–3.07, *p* = 0.002), which was not the case with the corresponding absolute value cutoff (HR: 1.03, 95% CI 0.67–1.58, *p* = 0.90, Figure S4)).

As the physiologic increase of sNfL in function of age is less pronounced in younger persons [7, 17], we tested whether, at least in this group, absolute sNfL values would identify patients at a higher risk of future relapses. However, we failed to demonstrate an association of absolute “high” versus “low” sNfL values with future relapses (HR: 1.26, 95% CI 0.87–1.84, *p* = 0.23, Figure S5), while the equivalent comparison with *Z* scores showed a substantially higher risk of future relapses (HR: 1.78, 95% CI 1.24–2.54, *p* = 0.002).

## 4 Discussion

We show that individuals with MS under fingolimod treatment and exhibiting “high” (i.e., ≥ Q4) age‐ and BMI‐adjusted sNfL *Z* scores have an increased risk for NEL within the subsequent year, as well as for relapses over the following 8 years, compared to those with “low” *Z* scores. This prognostic and, in terms of treatment efficacy, predictive capacity could not be demonstrated when using an according cutoff based on absolute concentrations of sNfL. This indicates that, different from group level analyses, the latter measure is insensitive for the interpretation of individual sNfL measures for monitoring treatment response and anticipating the future course of disease. In contrast, Z score‐based cutoffs match this purpose, and hence are a prerequisite for the use of sNfL in clinical practice, specifically in view of therapeutic decision, for example for therapy escalation.

Previously, it has been shown in group‐wise comparisons that the likelihood of elevated sNfL is higher with the concurrent presence of CEL on MRI [18, 19]. Our analysis shows that individuals with a “high” sNfL *Z* score, and with less magnitude those with a “high” absolute sNfL value, are at an increased risk of CEL compared to those with a “low” *Z* score or absolute value. Interestingly, there is a subgroup of patients without CEL whose sNfL levels were as high as in patients with CEL (by *Z* score or absolute value), confirming that sNfL is a more sensitive measure than CEL of present disease activity. Explanations for this observation are the fact that sNfL may capture neuronal damage that takes place outside of the anatomical structures covered by standard MRIs, for example, in the spinal cord, or where the MRI is insensitive to reveal ‘residual’ disease activity in relapsing–remitting MS and primary progressive MS [3, 20, 21].

The association between elevated baseline sNfL levels and the risk of future disease activity in persons with MS on the group level has also been the subject of previous studies, showing an increased risk within the following 3 months [22], the following year [23], or the following two years [21], while others do not report an association [24, 25]. Current results are consistent with our previous finding, which demonstrated that sNfL *Z* score outperformed absolute sNfL concentration in predicting evidence of disease activity (EDA‐3) within the subsequent year in MS patients [7]. However, these studies [21, 23, 24, 25] were mainly based on across‐group comparisons, that is, without reference to normative data that corrects for the physiologic change of sNfL levels for age, among other factors. In some of the previous studies, arbitrarily chosen absolute value cutoff levels of 9.3 pg/mL [24], 14.2 pg/mL [26], and 16 pg/mL [27] were used to define “high,” that is, pathologically elevated, sNfL levels. The different choice of cutoff levels in these studies may be due to factors like variations in assay methodology or the characteristics of the target population and led to a situation of limited cross‐comparability.

The cutoff of 10.8 pg/mL for absolute values used in the present study was selected on methodological grounds (≥ Q4 of sNfL levels) to allow an equal comparison between absolute concentrations and *Z* scores, based on comparable numbers above and below the cutoff. However, our findings are not specific to these cutoff values, and our study highlights that the advantage of *Z* scores over absolute concentrations, allowing meaningful conclusions of a sNfL measurement, is sustained over a wide range of selected thresholds. Importantly, the associations of sNfL *Z* score levels with clinical and MRI features of disease are fully congruent with earlier group‐wise comparisons mentioned above [7, 18, 19, 23, 24].

The group of MS patients below 55 years of age is here of specific interest, as here therapy escalation is nowadays a standard concept to improve the disease course in case of insufficient treatment response [7]. Overall, only a small number of patients are identifiable for having strongly increased levels of sNfL, based on absolute values, while 25% of patients had *Z* scores above 1.2 (88.5 percentile), many with levels below the absolute value cutoff (46/447: 13.7%). Based on the latter measure, they form a pool of patients misclassified by having “false negative” sNfL results, which may conflict with concomitant MRI findings, resulting in ambiguity over the current state of disease activity. Moreover, these patients may forfeit the potential to prevent future relapses by therapy escalation. Our results, therefore, corroborate the potential of sNfL *Z* scores along with clinical and MRI measures as a decision‐making tool for individual disease monitoring and prediction of treatment response, as emphasized in recent recommendations [28].

The use of *Z* scores, or percentiles as their equivalent, is routinely implemented in different disease areas where longitudinal assessments of biomarkers need to be normalized for physiological variability due to age or BMI, for example, in measuring the bone mineral density or in pediatrics for growth and body surface areas and echocardiographic evaluation of cardiac dimensions [29, 30, 31].

Our study comes with potential limitations. First, we used samples collected 1 year after treatment start, which may limit the generalizability of the results in case of shorter treatment duration. Second, our study was based on one sample for each patient; however, longitudinal assessments of sNfL have been shown to provide additional value for longer‐term disease activity monitoring [32] and the concept of *Z* scores also fully applies to such a setting. Third, our analysis does not include data on disease progression (i.e., progression independent of disease activity [PIRA]). Additionally, the technical validation of the applicability of *Z* scores across emerging analytical platforms is incomplete. However, efforts are underway to integrate all other major sNfL measurement platforms, whose values have recently been demonstrated to strongly correlate with those produced by Simoa [33]. Metrological projects aimed at standardizing sNfL measurements across platforms are also underway [34], but such projects may take years to complete.

In summary, our study emphasizes the importance of accounting for physiological variations associated with age and BMI to interpret individual sNfL results in people with MS with the highest possible accuracy. We recommend that clinicians seeking to use sNfL data for their assessment of treatment response in individual patients should utilize sNfL *Z* scores or percentiles rather than absolute values. Referring normative data for *Z* scores is accessible through an internet‐based app previously developed (https://shiny.dkfbasel.ch/baselnflreference/ [7]) for Simoa‐based measurements.

As current results are prognostic for disease course and predictive of the therapeutic effect of a DMT, *Z* scores increase the sensitivity to identify pathological values of sNfL not only in retrospective monitoring, but are as well applicable in prospective assessment of MS.

## Author Contributions

Jens Kuhle and Pascal Benkert had full access to all the data in the study and take responsibility for the integrity of the data and the accuracy of the data analysis. *Conception and design*: Maximilian Einsiedler, Aleksandra Maleska Maceski, Sofia Sandgren, Johanna Oechtering, David Leppert, Eline A.J. Willemse, Pascal Benkert, Jens Kuhle. *Acquisition, analysis, and/or interpretation of data*: Maximilian Einsiedler, Aleksandra Maleska Maceski, Sofia Sandgren, Johanna Oechtering, Sabine Schaedelin, Lisa Hofer, Amar Zadic, Juan Francisco Vilchez Gomez, Lester Melie‐Garcia, Alessandro Cagol, Riccardo Galbusera, Sebastian Finkener, Patrice Lalive, Marjolaine Uginet, Stefanie Müller, Caroline Pot, Amandine Mathias, Renaud Du Pasquier, Robert Hoepner, Andrew Chan, Giulio Disanto, Chiara Zecca, Marcus D'Souza, Lars G. Hemkens, Tobias Derfuss, Özgür Yaldizli, Patrick Roth, Claudio Gobbi, David Brassat, Björn Tackenberg, Henrik Zetterberg, Tjalf Ziemssen, Heinz Wiendl, Klaus Berger, Marco Hermesdorf, Fredrik Piehl, Ludwig Kappos, Cristina Granziera, Ahmed Abdelhak, David Leppert, Eline A.J. Willemse, Pascal Benkert, Jens Kuhle. *Drafting of the manuscript*: Maximilian Einsiedler, Aleksandra Maleska Maceski, Sofia Sandgren, Sabine Schaedelin, Lisa Hofer, David Leppert, Pascal Benkert, Jens Kuhle.

## Disclosure

Role of the funder/sponsor: The funding sources had no role in the design and conduct of the study; collection, management, analysis, and interpretation of the data; preparation, review, or approval of the manuscript; and decision to submit the manuscript for publication.

## Conflicts of Interest

Dr. Einsiedler reported receiving travel grants from Roche. Dr. Maleska Maceski reported no disclosures. Dr. Sandgren has received compensation for lectures and/or advisory board membership from Merck and Novartis. Dr. Oechtering reported receiving travel grants from Biogen Idec, Novartis, and Bayer; grants from Swiss MS Society; and serving on an advisory board for Roche. Dr. Schaedelin reported no disclosures. Ms. Hofer reported no disclosures. Mr. Zadic reported no disclosures. Mr. Vilchez Gomez reported no disclosures. Dr. Melie‐Garcia reported no disclosures. Dr. Cagol reported receiving grant support from the Horizon 2020 Eurostar program. Dr. Galbusera reported no disclosures. Dr. Finkener reported no disclosures. Dr. Lalive reported receiving speaker honoraria from Biogen Idec, Genzyme, Merck Serono, Novartis, Sanofi Aventis, and Teva; consulting fees from Biogen Idec, GeNeuro, Genzyme, Merck Serono, Novartis, Sanofi Aventis, and Teva; and research grants from Biogen Idec, Merck Serono, and Novartis. Dr. Uginet reported no disclosures. Dr. Müller reported receiving honoraria for travel, honoraria for lectures/consulting, and/or grants for studies from Almirall, Biogen, Celgene, Novartis, Teva, Merck Serono, Genzyme, Roche, and Bayer Schweiz. Dr. Pot reported receiving consulting fees and/or travel compensation, used exclusively for research support, for activities with Biogen, Merck, Novartis, Roche, and Sanofi Genzyme. Dr. Pot reported no disclosures. Dr. Mathias reported no disclosures. Dr. Du Pasquier reported no disclosures. Dr. Hoepner received speaker/advisor honorary from Merck, Novartis, Roche, Biogen, Alexion, Sanofi, Janssen, Bristol Myers Squibb, Teva/Mepha, and Almirall; he received research support within the last 5 years from Roche, Merck, Sanofi, Biogen, Chiesi, and Bristol Myers Squibb; he also received research grants from the Swiss MS Society, the SITEM Insel Support Fund, and is a member of the Advisory Board of the Swiss and International MS Society; he also serves as deputy editor in chief for Journal of Central Nervous System Disease and is part of the ECTRIMS Young Investigator Committee. Dr. Chan reported honoraria for boards/speaker activities from Actelion (Janssen/J&J), Alexion, Almirall, Biogen, Celgene (BMS), Genzyme, Horizon, Merck KGaA (Darmstadt, Germany), Novartis, Roche, Teva, all for hospital research funds; research support from Biogen, CSL Behring, Genzyme, Roche, and UCB; European Union, Swiss National Research Foundation. He reported being Associate Editor of the European Journal of Neurology, on the editorial board of Clinical and Translational Neuroscience, as well as topic editor of the Journal of International Medical Research. Dr. Disanto reported no disclosures. Dr. Zecca reported receiving honoraria for speaking/consulting fees or grants from Abbvie, Almirall, Biogen Idec, Celgene, Janssen, Genzyme, Lilly, Merck Serono, Novartis, Roche, Sanofi, and Teva Pharma outside the submitted work. Dr. D'Souza reported no disclosures. Dr. Hemkens reported no disclosures. Dr. Derfuss reported receiving speaker fees, research support, travel support, and/or serving on advisory boards, data safety monitoring boards, or Steering Committees of Actelion, Alexion, Celgene, Polyneuron, Novartis Pharma, Merck Serono, Sanofi, Biogen, Teva, Bayer Pharma, GeNeuro, Mitsubishi Pharma, MedDay, Roche, and Genzyme. Dr. Yaldizli reported receiving grants from the European Committee for Treatment and Research in Multiple Sclerosis/European Magnetic Resonance Imaging in Multiple Sclerosis network, the University of Basel, Pro Patient Stiftung University Hospital Basel, Free Academy Basel, the Swiss Multiple Sclerosis Society, and advisory board/lecture and consultancy fees from Roche, Sanofi Genzyme, Almirall, Biogen, and Novartis. Dr. Roth has received honoraria for lectures or advisory board participation from Alexion, Bristol Myers Squibb, Boehringer Ingelheim, Debiopharm, Galapagos, Merck Sharp and Dohme, Laminar, Midatech Pharma, Novocure, QED, Roche, Sanofi, and Servier and research support from Merck Sharp and Dohme and TME Pharma. Dr. Gobbi reported receiving honoraria for speaking/consulting or grants from Abbvie, Almirall, Biogen Idec, Celgene, Genzyme, Merck Serono, Novartis, Roche, Teva Pharma. Dr. Brassat reported being a current Novartis employee. Dr. Tackenberg reported other funding from F. Hoffmann‐La Roche outside the submitted work; and is a full‐time employee of F. Hoffmann‐La Roche. Dr. Zetterberg is a Wallenberg Scholar and a Distinguished Professor at the Swedish Research Council supported by grants from the Swedish Research Council (#202300356, #202201018 and #201902397), the European Union's Horizon Europe research and innovation program under grant agreement No 101053962, Swedish State Support for Clinical Research (#ALFGBG71320), the National Institute for Health and Care Research University College London Hospitals Biomedical Research Centre, and the UK Dementia Research Institute at UCL (UKDRI1003); he has served on scientific advisory boards and/or as a consultant for Abbvie, Acumen, Alector, Alzinova, ALZpath, Amylyx, Annexon, Apellis, Artery Therapeutics, AZTherapies, Cognito Therapeutics, CogRx, Denali, Eisai, Enigma, LabCorp, Merry Life, Nervgen, Novo Nordisk, Optoceutics, Passage Bio, Pinteon Therapeutics, Prothena, Quanterix, Red Abbey Labs, reMYND, Roche, Samumed, Siemens Healthineers, Triplet Therapeutics, and Wave, has given lectures sponsored by Alzecure, BioArctic, Biogen, Cellectricon, Fujirebio, Lilly, Novo Nordisk, Roche, and WebMD, and is a cofounder of Brain Biomarker Solutions in Gothenburg AB (BBS), which is a part of the GU Ventures Incubator Program. Dr. Ziemssen reported no disclosures. Dr. Wiendl reported receiving honoraria for acting as a member of scientific advisory boards from Abbvie, Alexion, Argenx, Bristol Myers Squibb/Celgene, Janssen, Merck, and Novartis; speaker honoraria and travel support from Alexion, Biogen, Bristol Myers Squibb, F. Hoffmann‐La Roche Ltd., Genzyme, Merck, Neurodiem, Novartis, Roche Pharma AG, Teva Pharma, and WebMD Global; consultant fees from Abbvie, Actelion, Argenx, Biogen, Bristol Myers Squibb, EMD Serono, Fondazione Cariplo, Gossamer Bio, Idorsia, Immunic, Immunovant, Janssen, Lundbeck, Merck, NexGen, Novartis, PSI CRO, Roche, Sanofi, Swiss Multiple Sclerosis Society, UCB, and Worldwide Clinical Trials; and research funding from the German Ministry for Education and Research, Deutsche Forschungsgesellschaft, Deutsche Myasthenie Gesellschaft e.V., Alexion, Amicus Therapeutics Inc., Argenx, Biogen, CSL Behring, F. Hoffmann‐La Roche, Genzyme, Merck KgaA, Novartis Pharma, Roche Pharma, and UCB Biopharma. Dr. Berger reported receiving a grant from the German Ministry of Education and Research (within the German Competence Net Multiple Sclerosis) plus additional funds from Biogen, all to the University of Münster, for an investigator‐initiated adverse event registry for patients with multiple sclerosis. Dr. Hermesdorf reported no disclosures. Dr. Piehl reported receiving grants from Merck KGaA and UCB; data safety monitoring board member fees from Chugai, Lundbeck, and Roche; and fees for the preparation of expert witness statements from Novartis outside the submitted work. Dr. Kappos' employer (University Hospital Basel) has received and dedicated to research support fees for board membership, consultancy or speaking, or grants in the past 3 years from Abbvie, Actelion, Advancell, Allozyne, Auriga Vision AG, Bayer, Bayhill, Biogen Idec, BioMarin, Celgene, CSL Behring, dfmp Molnia & Pohlman, Eisai, Eli Lilly EU, EMD Serono, Genentech, Genmab, GeNeuro SA, Genzyme, Gianni Rubatto Foundation, Glaxo Smith Kline, Glenmark, Innosuisse, Janssen, Japan Tobacco, Merck Serono, MediciNova, Minoryx Therapeutics, Mitsubishi Pharma, MH Consulting, Neurostatus‐UHB AG, Novartis, Novartis Research Foundation, Novo Nordisk, Österreichische Gesellschaft für Neurologie, Peptimmune, Roche, Roche Research Foundation, Santhera, SanofiAventis, Senda Biosciences Inc., Swiss MS Society, Swiss National Research Foundation, Teva Pharmaceutical Industries Ltd., TG Therapeutics, UCB, Wellmera AG, and Wyeth; and Dr. Kappos reported having a patent for Neurostatus‐UHB AG with royalties paid to institution (University Hospital Basel); being CEO (employment by University Hospital Basel, MAGNIMS Steering Committee) and a board member of the European Charcot Foundation. Dr. Granziera reported The University Hospital Basel (USB), as the employer of C.G., has received the following fees which were used exclusively for research support: (1) advisory boards and consultancy fees from Actelion, Novartis, GenzymeSanofi, GeNeuro, Hoffmann‐La Roche and Siemens Healthineers; (2) speaker fees from Biogen, Hoffmann‐La Roche, Teva, Novartis, Janssen and GenzymeSanofi; and (3) research grants from Hoffmann‐La Roche, GeNeuro, Genzyme, Biogen. Dr. Abdelhak reported receiving research grants from the German Multiple Sclerosis Society, Roche, and Denali Therapeutics. Dr. Leppert reported having been chief medical officer of GeNeuro until the end of 2024. Dr. Benkert reported no disclosures. Dr. Kuhle reported receiving grants from Swiss National Science Foundation, Swiss MS Society, Biogen, Celgene, Merck, Novartis, Roche, Sanofi, Progressive MS Alliance, University of Basel, Octave Bioscience.

## Supporting information


Data S1.

## Data Availability

Written requests for access to the data reported in this paper will be considered by the corresponding author and the Scientific Board of the Swiss MS Cohort Study, and a decision will be made about the appropriateness of the use of the data. If the use is appropriate, a data sharing agreement will be put in place before a fully de‐identified version of the dataset used for the analysis with individual participant data is made available.
