# Supplementary material for: Serum Neurofilament Light Chain in Multiple Sclerosis: Superiority of Age‐ and BMI‐Corrected Z Scores/Percentiles Over Absolute Cutoff Values for Prediction of Treatment Response
Source: Ann Clin Transl Neurol. 2025 Aug 3;12(11):2214–25. doi: 10.1002/acn3.70149 (PMC12623839; doi:10.1002/acn3.70149)
Supplement: Supplementary file 1 — Data S1. [file ACN3-12-2214-s001.docx]

**Serum neurofilament light chain in multiple sclerosis: superiority of age- and BMI-corrected Z scores/percentiles over absolute cut-off values for prediction of treatment response**

Einsiedler M, Maleska Maceski A, Sandgren S et al.

**Online-only supplements**

**Index:** Page

**eTables:**

# 1. eTable 1. 2 Comparison of absolute values cut-off and sNfL Z score cut-off.

# 2. eTable 2. 3 Estimated proportion of patients with relapse at different timepoints

# of follow-up (Kaplan-Meier estimates) stratified by “high” or “low” sNfL Z score

# at index sample

# 3. eTable 3. 4 Patient characteristics at start of oral DMT for generalizability analysis

**eFigures:**1. eFigure 1. 5
Differences in absolute values and Z scores of sNfL according to
presence of contrast enhancing lesions (CEL).

2. eFigure 2. 6
Differences in absolute values and in Z scores of sNfL according to past or future new/enlarging T2w lesions (NEL).

3. eFigure 3. 7

Time to first relapse depending on sNfL levels one year after DMT start

using either absolute values or Z scores
- cohort including patients having started various oral DMTs

4. eFigure 4. 8
Time to first relapse depending on sNfL levels one year after fingolimod start

using either absolute values or Z scores

- sensitivity analysis censoring patients at DMT switch or treatment interruption.

5. eFigure 5. 9

Time to first relapse depending on sNfL levels one year after fingolimod start

using either absolute values or Z scores

- sensitivity analysis restricted to patients younger than 55 years at index sample.

**eTable 1. Comparison of cut-offs for absolute values and Z score of sNfL.**

| **sNfL Z Score**  **Absolute value of sNfL** | **High (≥1.2)** | **Low (<1.2)** | **Total** |
| --- | --- | --- | --- |
| **High (≥10.8 pg/ml)** | 68 (15%) | 44 (10%) | 112 (25%) |
| **Low (<10.8 pg/ml)** | 46 (10%) | 289 (65%) | 335 (75%) |
| **Total** | 114 (25%) | 333 (75%) | 447 (100%) |

Abbreviations: sNfL: serum neurofilament light chain.
Results are presented as n (%).

**eTable 2. Estimated proportion of patients with relapse at different timepoints of follow-up (Kaplan-Meier estimates) stratified by “high” or “low” sNfL Z score at index sample.**

| **Years of follow-up after index sampling** | **Proportion of relapses [95% CI]** | |
| --- | --- | --- |
|  | **High sNfL Z score^a^** | **Low sNfL Z score^b^** |
| **1** | 24.9% [16.4, 32.4] | 6.3% [3.7, 8.9] |
| **2** | 32.2% [23.0, 40.4] | 12.3% [8.6, 15.8] |
| **3** | 36.2% [26.5, 44.6] | 17.2% [12.9, 21.2] |
| **4** | 39.2% [29.3, 47.7] | 22.3% [17.5, 26.8] |
| **5** | 40.3% [30.3, 48.9] | 25.2% [20.2, 29.9] |

^a^ High sNfL Z score was defined as ≥1.2 (quartile 4).
^b^ Low sNfL Z score was defined as <1.2 (quartiles 1-3).

Abbreviations: sNfL: serum neurofilament light chain; CI: confidence interval.

**eTable 3. Patient characteristics at start of oral DMT for patients who had started treatment with fingolimod, dimethyl fumarate, teriflunomide, diroximel fumarate, ozanimod or siponimod.**

|  | n=713 |
| --- | --- |
| Sex (female) | 468 (65.6) |
| Age, y | 41.9 [33.3, 50.8] |
| EDSS | 2.0 [1.5, 3.0] |
| Disease duration, y | 7.6 [3.0, 14.2] |
| Relapse <3 months before treatment start | 147 (20.6) |
| Treatment start to index sample, y | 1.0 [0.9, 1.3] |
| Follow-up duration after index sample, y | 7.5 [4.3, 10.5] |
| At index sample |  |
| sNfL (pg/ml) | 7.8 [5.5, 11.0] |
| sNfL Z score | 0.3 [-0.6, 1.1] |
| DMT |  |
| Fingolimod | 447 (62.7) |
| Dimethyl fumarate | 186 (26.1) |
| Teriflunomide | 59 (8.3) |
| Diroximel fumarate | 12 (1.7) |
| Ozanimod | 6 (0.8) |
| Siponimod | 3 (0.4) |

Variables are expressed as n (%) or median [IQR].
DMT: disease modifying treatment, EDSS: Expanded Disability Status Scale, IQR: interquartile range, sNfL: serum neurofilament light chain, y: years.

**eFigure 1. Differences in (A) absolute values and (B) Z scores of sNfL according to presence of contrast enhancing lesions.**


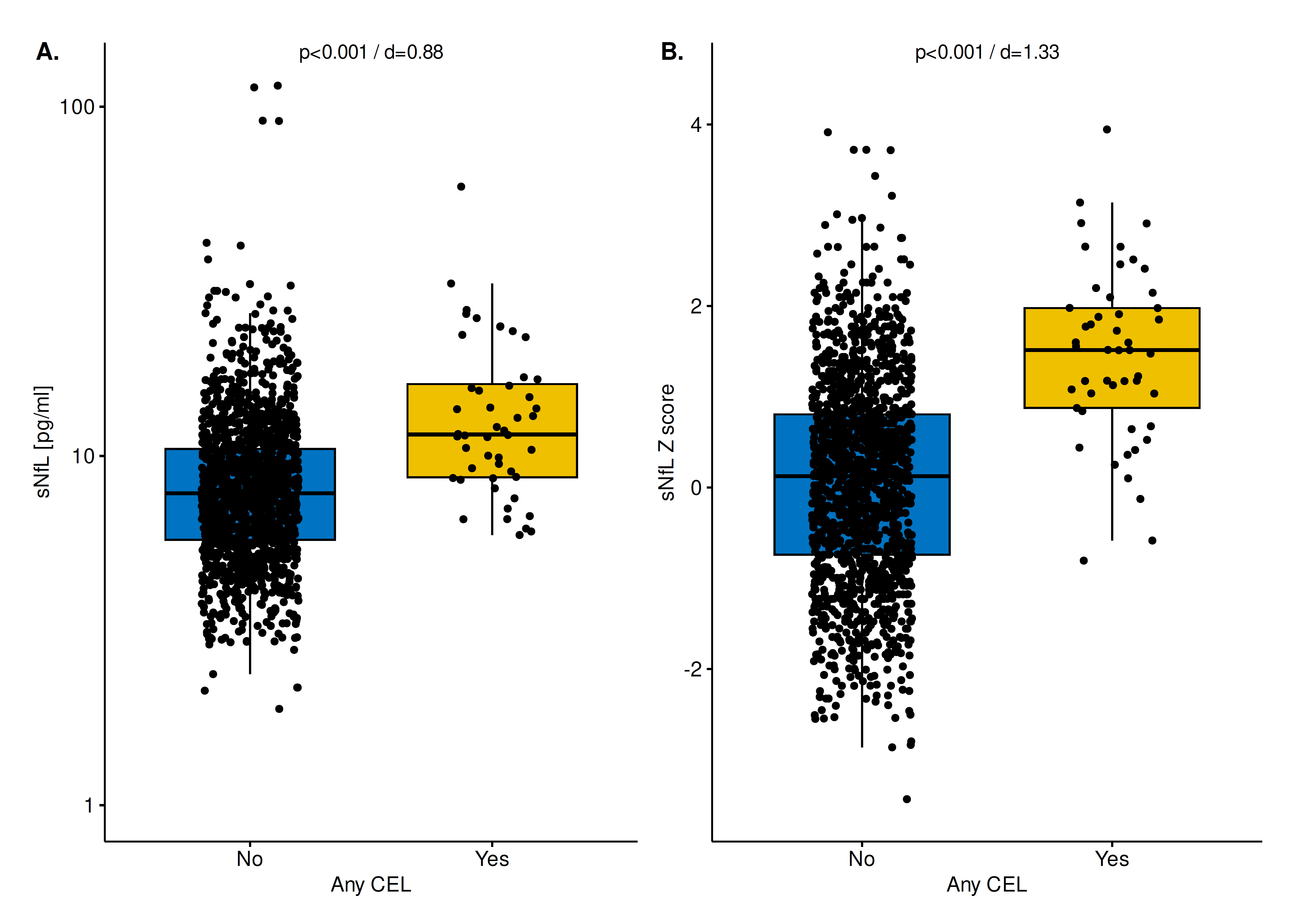


Legend:

**A.** Absolute sNfL was increased by 53% (estimate 1.53, 95 % CI 1.27-1.85, p<0.001, *d*=0.88) in patients with versus without CELs

**B**. sNfL Z scores were increased by 0.95 units (95 % CI 0.63-1.26, p<0.001, *d*=1.33) in patients with versus without CELs.

Estimates from univariable linear GEE models with sNfL as dependent variable and CELs (dichotomized in presence versus absence) as independent variable.

1420 sampling timepoints with available MRI information included after start of fingolimod treatment.

Application of sNfL Z scores versus absolute sNfL concentrations increased the magnitude of difference (as expressed by the effect size measure Cohen’s d).

Abbreviations: CEL: contrast enhancing lesion, CI: confidence interval, GEE: generalized estimating equation, sNfL: serum neurofilament light chain.

**eFigure 2. Differences in absolute values and in Z scores of sNfL according to (A) past or (B) future new/enlarging T2w lesions.**


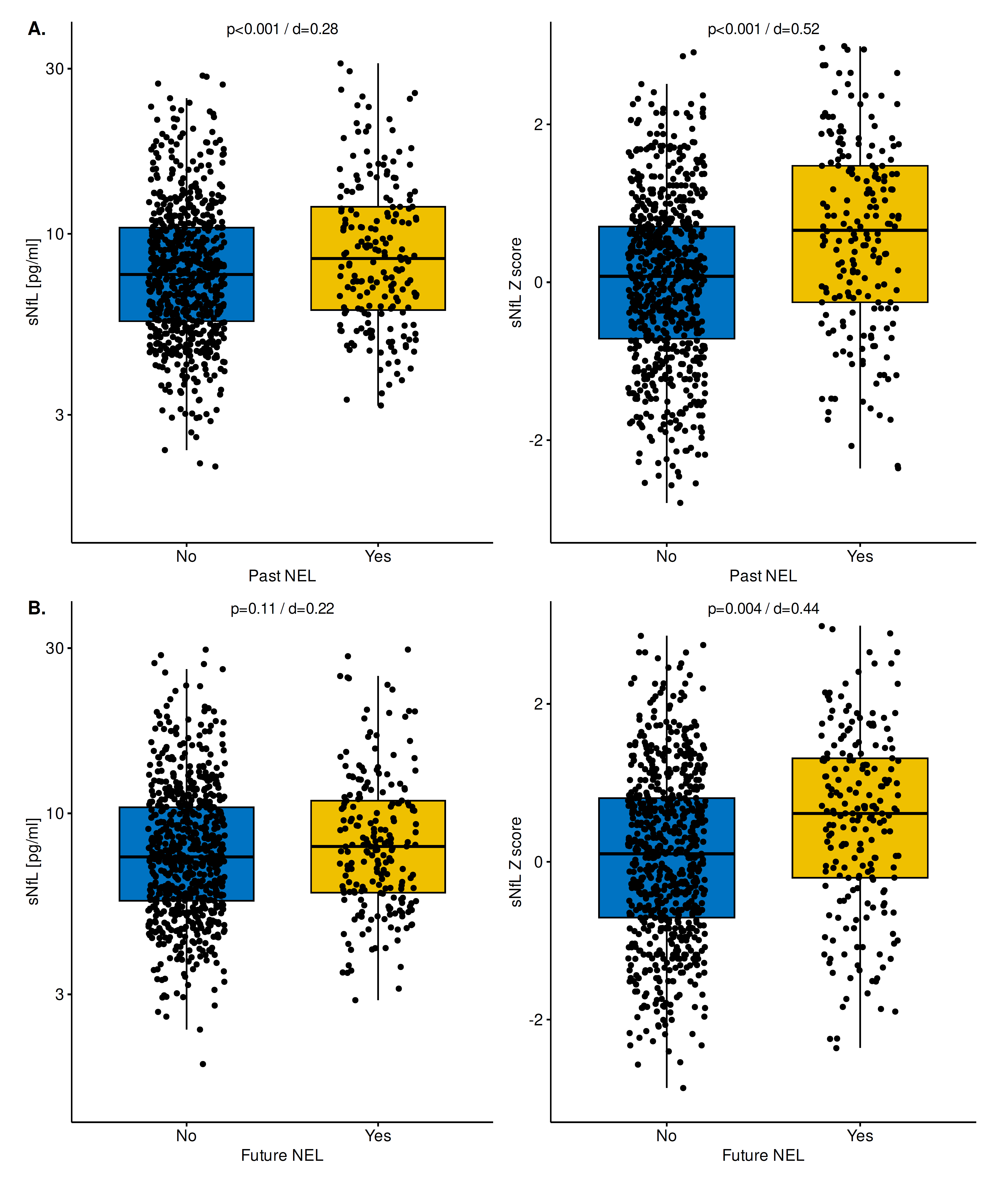


Legend:

**A.** In patients with past NEL (n=193, 21.5 % of 899 timepoints with available MRI information), absolute sNfL values were higher compared to patients with stable MRI (median [IQR] 8.5 pg/ml [6.1, 12.5] versus 7.6 [5.6, 10.4], est. 1.15, 95%CI 1.07-1.23, p<0.001, *d*=0.28). This difference was more pronounced for sNfL Z scores (0.67 [-0.25, 1.48] versus 0.08 [-0.73, 0.71], est. +0.45 Z score units, 95%CI 0.28-0.61, p<0.001, *d=*0.52).

**B.** In patients with future NEL (n=191, 21.8% of 878 timepoints), absolute sNfL values were not different compared to patients with stable MRI (8.1 pg/ml [6.0, 11.3] versus 7.5 pg/ml [5.6, 10.5], est. 1.06, 95%CI 0.99-1.13, p = 0.11, *d*=0.22), whereas sNfL Z scores were higher (0.64 [-0.18, 1.36] versus 0.13 [-0.71, 0.84], est. +0.26 Z score units, 95%CI 0.09-0.44, p = 0.004, *d*=0.44).
Application of sNfL Z scores versus absolute sNfL concentrations increased the magnitude of difference (as expressed by the effect size measure Cohen’s d).
Estimates from univariable linear GEE models with sNfL as dependent variable and future and past NELs (separately dichotomized in presence versus absence) as independent variables in separate models.

Abbreviations: CI: confidence interval, MRI: magnetic resonance imaging, NEL: new/enlarging T2w lesions, sNfL: serum neurofilament light chain, T2w: T2-weighted.

**eFigure 3. Time to first relapse depending on sNfL levels one year after DMT start using either (A) absolute values or (B) Z scores –cohort including patients having started various oral DMTs**


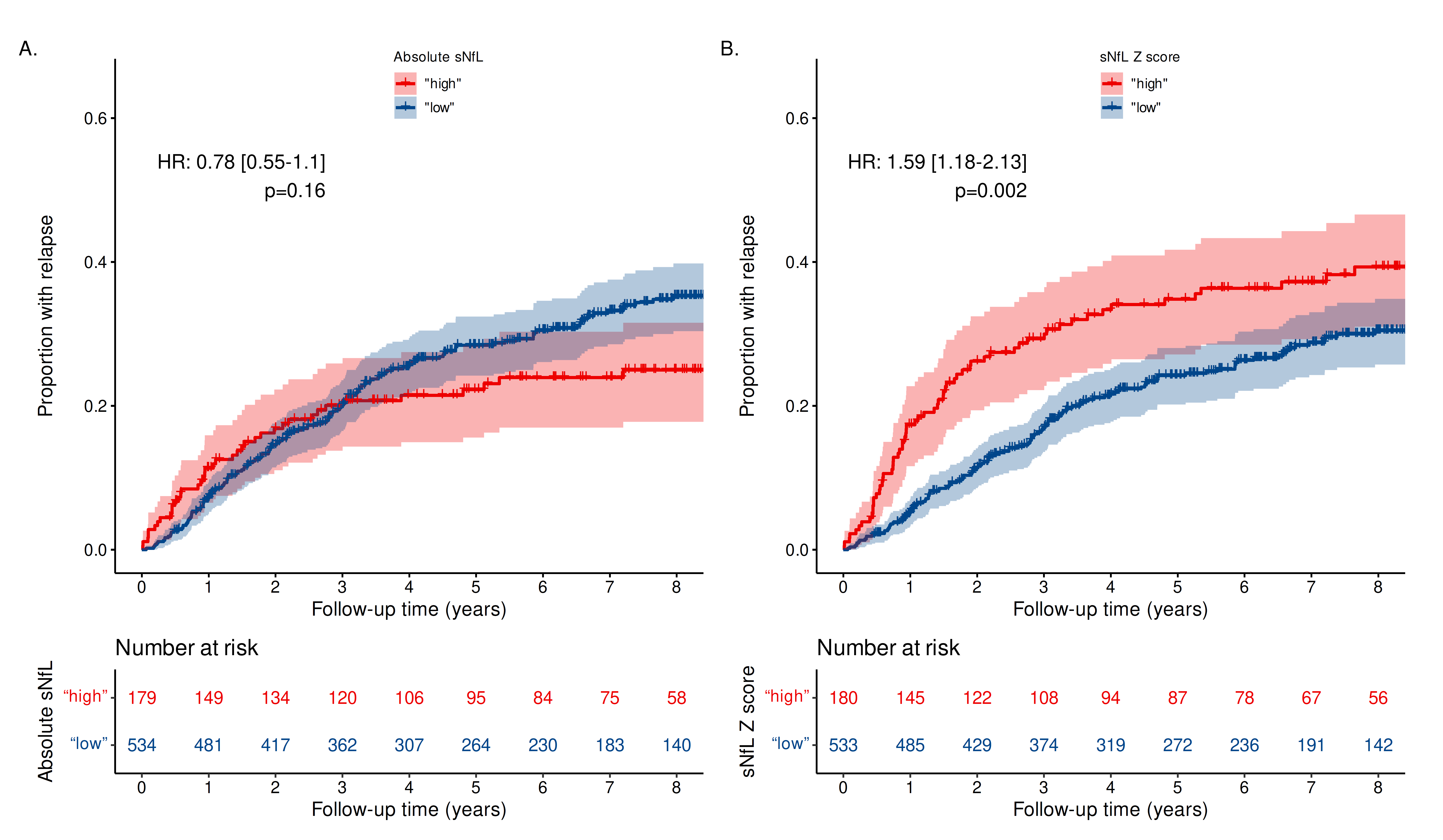


Legend:

**A.** Time to first relapse comparing “high” absolute sNfL (Q4; ≥11.0 pg/ml) to “low” sNfL (Q1-3; <11.0 pg/ml).

**B.** Time to first relapse comparing “high” sNfL Z score (Q4; ≥1.1) to “low” sNfL (Q1-3; <1.1).

Using a cut-off with sNfL Z score outperformed absolute sNfL values in predicting relapses one year after start of fingolimod (absolute sNfL: HR: 0.78, 95%CI 0.55-1.10, p=0.16; sNfL Z scores: HR: 1.59, 95%CI 1.18-2.13, p=0.002)

Abbreviations: CI: confidence interval, DMT: disease-modifying therapy, HR: hazard ratio, Q: quartile; sNfL: serum neurofilament light chain

**eFigure 4. Time to first relapse depending on sNfL levels one year after fingolimod start using either (A) absolute values or (B) Z scores - sensitivity analysis censoring patients at DMT switch.**


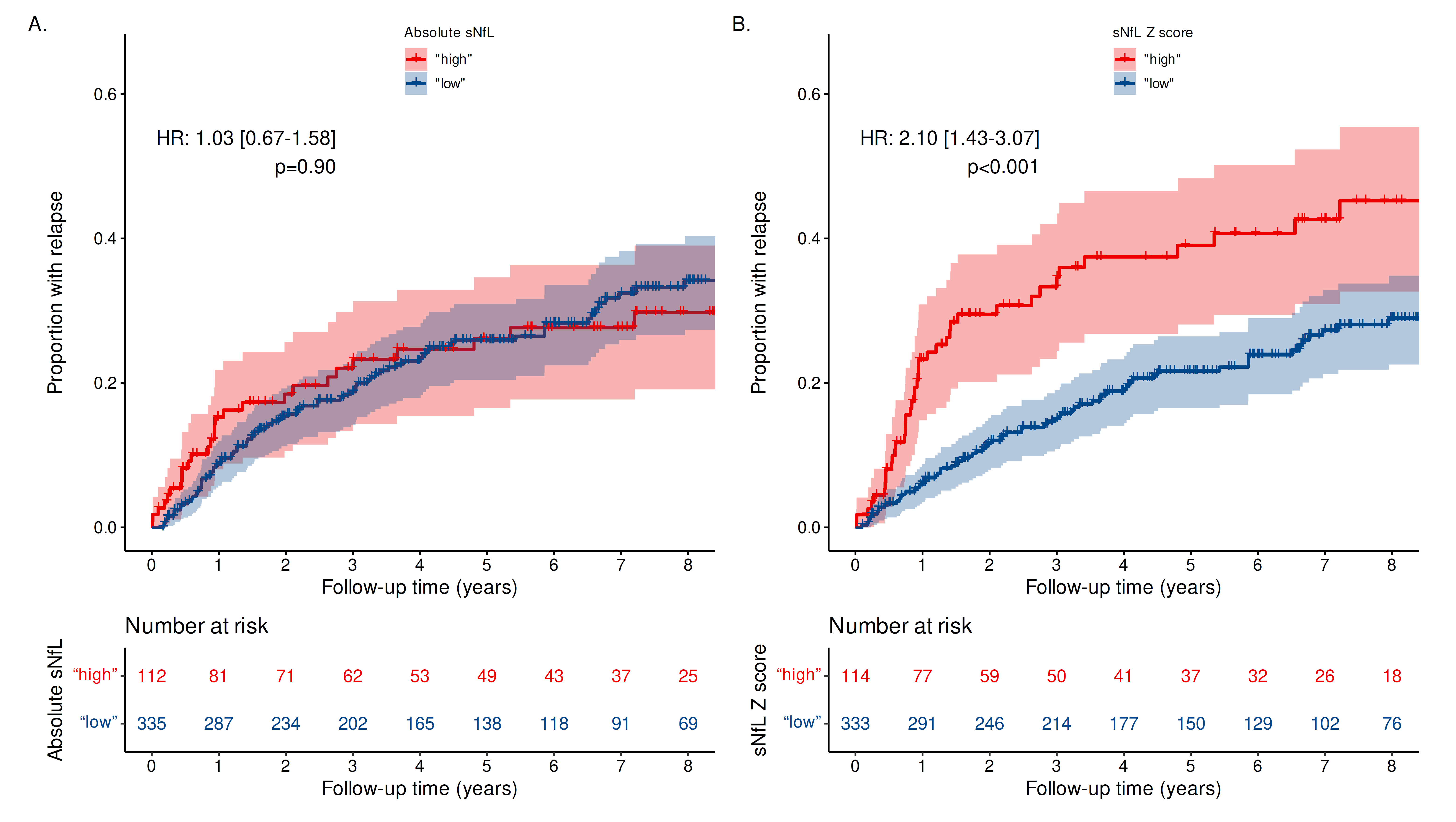


Legend:

**A.** Time to first relapse comparing “high” absolute sNfL (Q4; ≥10.8 pg/ml) to “low” sNfL (Q1-3; <10.8 pg/ml).

**B.** Time to first relapse comparing “high” sNfL Z score (Q4; ≥1.2) to “low” sNfL (Q1-3; <1.2).

Using a cut-off with sNfL Z score outperformed absolute sNfL values in predicting relapses one year after start of fingolimod (absolute sNfL: HR: 1.03, 95%CI 0.67-1.58, p=0.90; sNfL Z scores: HR: 2.10, 95%CI 1.43-3.07, p<0.001)

Abbreviations: CI: confidence interval, DMT: disease-modifying therapy, HR: hazard ratio, Q: quartile; sNfL: serum neurofilament light chain.

**eFigure 5. Time to first relapse depending on sNfL levels one year after fingolimod start using either (A) absolute values or (B) Z scores - sensitivity analysis restricted to patients younger than 55 years at index sample.**


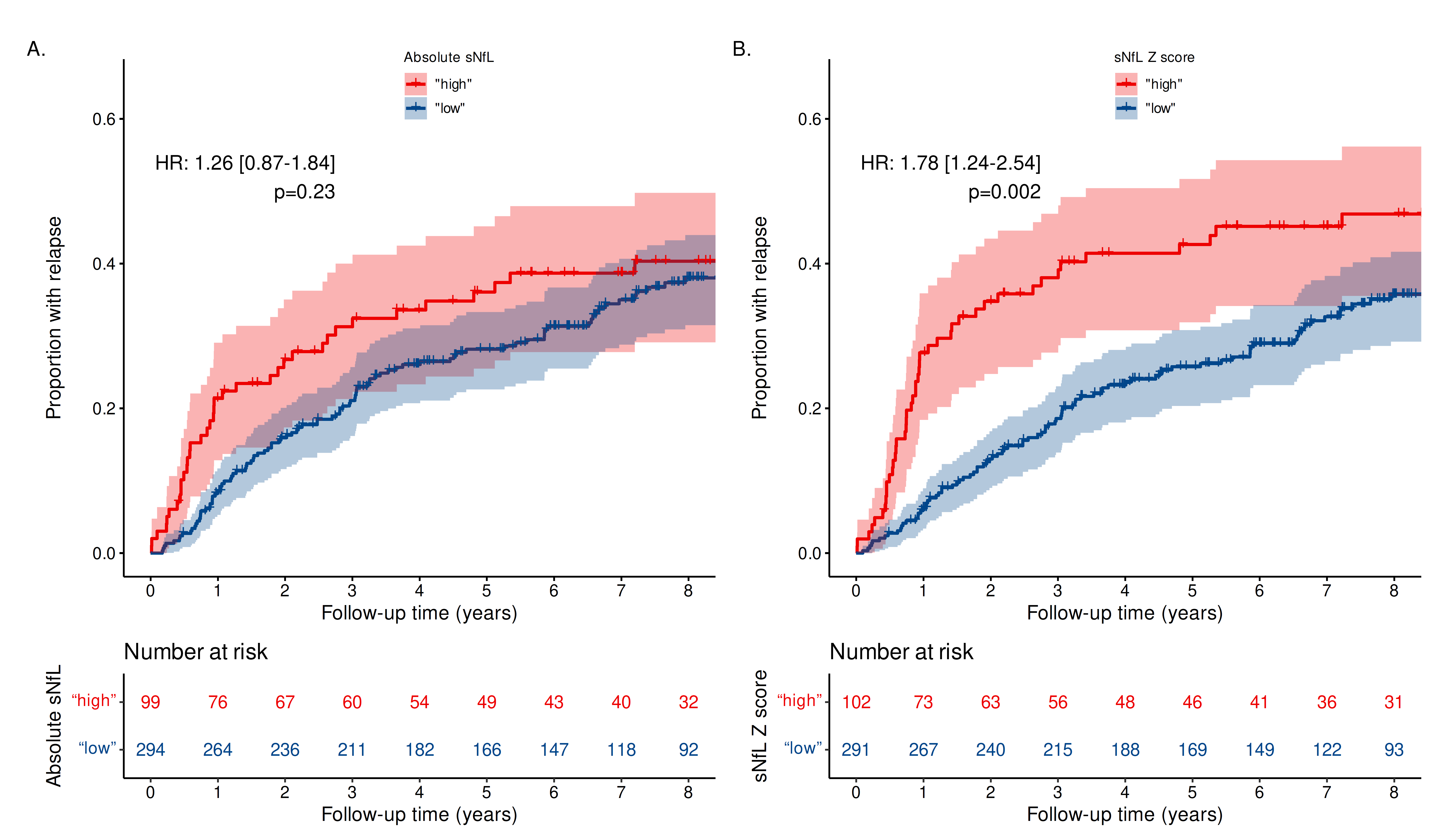


Legend:

**A.** Time to first relapse comparing “high” absolute sNfL (Q4; ≥9.9 pg/ml) to “low” sNfL (Q1-3; <9.9 pg/ml).

**B.** Time to first relapse comparing “high” sNfL Z score (Q4; ≥1.3) to “low” sNfL (Q1-3; <1.3)

Using a cut-off with sNfL Z score outperformed absolute sNfL values in predicting relapses one year after start of fingolimod, even in younger patients (absolute sNfL: HR: 1.26, 95%CI 0.87-1.84, p=0.23; sNfL Z scores: HR: 1.78, 95%CI 1.24-2.54, p=0.002).

Abbreviations:

CI: confidence interval, HR: hazard ratio, Q: quartile, sNfL: serum neurofilament light chain
